# An Evaluation of the Accuracy and Performance of Lightweight GPS Collars in a Suburban Environment

**DOI:** 10.1371/journal.pone.0068496

**Published:** 2013-07-09

**Authors:** Amy L. Adams, Katharine J. M. Dickinson, Bruce C. Robertson, Yolanda van Heezik

**Affiliations:** 1 Department of Zoology, University of Otago, Dunedin, New Zealand; 2 Department of Botany, University of Otago, Dunedin, New Zealand; Pacific Northwest National Laboratory, United States of America

## Abstract

The recent development of lightweight GPS collars has enabled medium-to-small sized animals to be tracked via GPS telemetry. Evaluation of the performance and accuracy of GPS collars is largely confined to devices designed for large animals for deployment in natural environments. This study aimed to assess the performance of lightweight GPS collars within a suburban environment, which may be different from natural environments in a way that is relevant to satellite signal acquisition. We assessed the effects of vegetation complexity, sky availability (percentage of clear sky not obstructed by natural or artificial features of the environment), proximity to buildings, and satellite geometry on fix success rate (FSR) and location error (LE) for lightweight GPS collars within a suburban environment. Sky availability had the largest affect on FSR, while LE was influenced by sky availability, vegetation complexity, and HDOP (Horizontal Dilution of Precision). Despite the complexity and modified nature of suburban areas, values for FSR (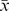 = 90.6%) and LE (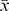 = 30.1 m) obtained within the suburban environment are comparable to those from previous evaluations of GPS collars designed for larger animals and within less built-up environments. Due to fine-scale patchiness of habitat within urban environments, it is recommended that resource selection methods that are not reliant on buffer sizes be utilised for selection studies.

## Introduction

The development of Global Positioning System (GPS) technologies in the mid-1990s has enabled the use of GPS telemetry to investigate habitat and resource selection, space use, and movement patterns of wildlife [1], [2]. GPS telemetry overcomes many of the disadvantages of traditional VHF (Very High Frequency) radio-tracking, as more accurate locations can be continuously collected regardless of season, time of day, weather conditions, and terrain without the need for fieldworkers. It also avoids the problem of animals modifying their behaviour due to proximity to humans [3], [4].

Despite the clear advantages, the GPS receiver is subject to two types of error: receivers may fail to acquire the necessary satellite signals over a pre-defined time schedule (missed data; termed fix success rate (FSR)), and locations acquired may be spatially inaccurate (referred to as location error (LE)). LE can result in misclassification of habitats and/or resources in selection studies, leading to poor management decisions regarding species and/or habitat management [5], [6], [7]. These two types of error are influenced by numerous environmental and technological factors that can affect signal transmission from satellites to receivers [8].

The main environmental factors affect FSR and LE of GPS collars by obstructing or reflecting the transmission of satellite signals and include topography [6], [9], [10] and vegetation characteristics, particularly those associated with species composition and structural complexity (principally stem density, life form, canopy height and cover) [4], [6], [10], [11], [12], [13], [14], [15], [16], [17], [18], [19].

Technological factors influencing FSR and LE include the number of satellites present and their geometric configuration [11], [20]. A three-dimensional (3-D) positional fix is obtained when signals from four or more satellites are used, while two-dimensional (2-D) positional fixes are acquired from three satellites [11], [16]. Generally, 3-D fixes are more accurate than 2-D fixes due to the higher number of satellites used to acquire the fix [11], [16]. The geometric configuration of available satellites is represented by the Dilution of Precision (DOP, e.g. horizontal (HDOP) or vertical (VDOP)). Low DOP values are acquired when satellites are spaced widely apart, resulting in smaller triangulation errors and more accurate positional fixes (i.e. low LEs) than fixes associated with high DOP values (representing poor satellite geometry) [7], [16], [21]. Other technical factors influencing FSR and LE include weakening GPS batteries [22], differences in collar brands, malfunctioning electronics, errors in satellite clocks, and multipath signals. The latter occurs when the GPS collar receiver acquires multiple satellite signals due to reflection off nearby surfaces [16], [21], [23], [24].

The magnitude of error surrounding a GPS receiver in any given environment can be estimated and related to environmental and technical variables [25]. Therefore, researchers using GPS radio-telemetry on wildlife should first evaluate the error associated with their GPS device within their specific study habitat [26]. The performance of GPS collars designed for large animals has been widely evaluated in both field trials and stationary tests performed at known locations [9], [11], [16], [20]. However, only a handful of studies have examined the performance of lightweight GPS collars for use on small-to-medium sized animals [14], [27], [28], [29], which require their own testing, as the smaller technological GPS components may result in differences in how these GPS collars operate compared to larger collars [28].

Most research evaluating the performance of GPS collars of varying size has been conducted in non-urban environments [7], [9], [11], [14], [16], [20], [23], [28], [30], [31]. Only one study has investigated the performance of a small GPS receiver (29–36 g for deployment on feral pigeons) within an urban environment in the central part of a major industrial city (Basel, Switzerland; [29]). Urban environments are highly heterogeneous: on a broad scale landscape components include industrial, commercial, and residential areas as well as parks, reserves, and waste land, while on a fine scale there can be significant patchiness within these landscape components, with buildings and vegetation varying in height and density. Vegetative surfaces have already been shown to affect GPS error by blocking or reflecting satellite signals within natural habitats [13], and this multipath error is likely to be greater in urban environments due to the presence of buildings. A large proportion of the urban landscape is residential (between 20% and 26% of large cities [32] and 36% in a smaller city [33]), and these suburban habitats have been shown to support significant populations of wildlife [34]. Evaluation of the performance and accuracy of GPS collars within suburban areas is therefore necessary to provide information about the error associated with collected fixes to enable data correction, or the implementation of appropriate buffers in selection studies that occur in suburban habitat [8]. Incorporating error information within a study site can help minimise the potential of habitat/resource misclassification, and reduce incorrect conclusions regarding resource selection, and subsequent poor management decisions.

This study aimed to evaluate the main environmental and technical factors causing error (FSR and LE) in lightweight GPS collars using stationary tests across a typical suburban environment as a precursor to research on resource selection of the common brushtail possum (*Trichosurus vulpecula*; hereafter referred to as possum). We aimed to produce an error estimate specifically for suburban habitats for use in selection studies. Three main environmental variables were predicted *a priori* to influence GPS collar performance in this environment: suburban areas which differ in the complexity of vegetation present within a garden; the degree of canopy closure (referred to here as sky availability); and proximity to buildings. Technical factors predicted *a priori* to influence collar performance included the number of satellites and satellite geometry (HDOP).

## Methods

### Ethics Statement

This study was conducted with permission from householders to conduct stationary GPS collar tests within their gardens.

### Study Area

Collar performance was tested in suburban gardens of Dunedin, New Zealand (45°52′S, 170°30′E), with properties typically consisting of detached single or double storey houses, and property sizes ranging between 0.018 ha and 0.282 ha (median = 0.061; mean = 0.075±0.01 SE; van Heezik, unpublished data). Houses are typically surrounded by vegetation, usually on all sides, covering between 15% and 95% of the total area of the property (median = 62%; van Heezik, unpublished data). To gain an understanding of factors influencing collar performance in the suburban environment, sample sites were randomly selected from properties where subsequent GPS collaring of possums was to occur. Suburban habitats fall into three distinguishable categories (Res 1, Res 2, Res 3; Table 1; [35], [36]) according to variations in housing densities, garden structures, and vegetation complexity. The properties selected represented this spectrum of suburban residential development and would be typical of suburbs in most cities.

**Table 1:** Descriptions of the three suburban habitat types in which lightweight GPS collars were evaluated in, Dunedin, New Zealand, as defined by Freeman and Buck [34].

Habitat Type	Habitat Description
Res 1	Residential areas with greater than one third of the property size comprised of mature, structurally-complex gardens containing an assortment of lawns, hedges, shrubs, and large established trees. Green cover totals 70% with a mean housing density of 11.6/ha (SD = 1.98, n = 14) [35].
Res 2	Residential areas with greater than one third of the property size comprised of structurally-less complex gardens dominated by lawns. Green cover ranges between 42–50% with a mean housing density of 12.52/ha (SD = 2.27, n = 20 suburbs) [35].
Res 3	Residential areas with no garden or where less than one third of the property is garden dominated by flowerbeds or lawn. Green cover totals 30% with a mean housing density of 28.6/ha (SD = 3.14, n = 6 suburbs) [35].

### Stationary Collar Tests

Three 120 g Wildlife GPS data-logger collars (Sirtrack Electronics, Havelock North, New Zealand, http://www.sirtrack.com) equipped with a 12-channel GPS receiver Trimble iQ to be later deployed on possums within a suburban environment were tested. At each site, collars were configured to acquire a location every 15 minutes in one 24 hour period, which encompasses two complete satellite constellation cycles incorporating all possible satellite configurations (97 possible fixes) [17], [21]. Data were stored in a built-in memory capable of storing 40,000 fixes until collar retrieval, and included information such as the date, time, longitude, latitude, number of satellites present, and HDOP for each successful fix. Altitude was not directly measured by these GPS collars.

Firstly, to verify that all test collars were operating similarly, the three collars were deployed simultaneously at a known survey mark under open sky to assess the performance and accuracy of each collar. Differences between the FSR and LE values between the three collars were quantified using one-way analysis of variance (ANOVA).

**Figure 1:**
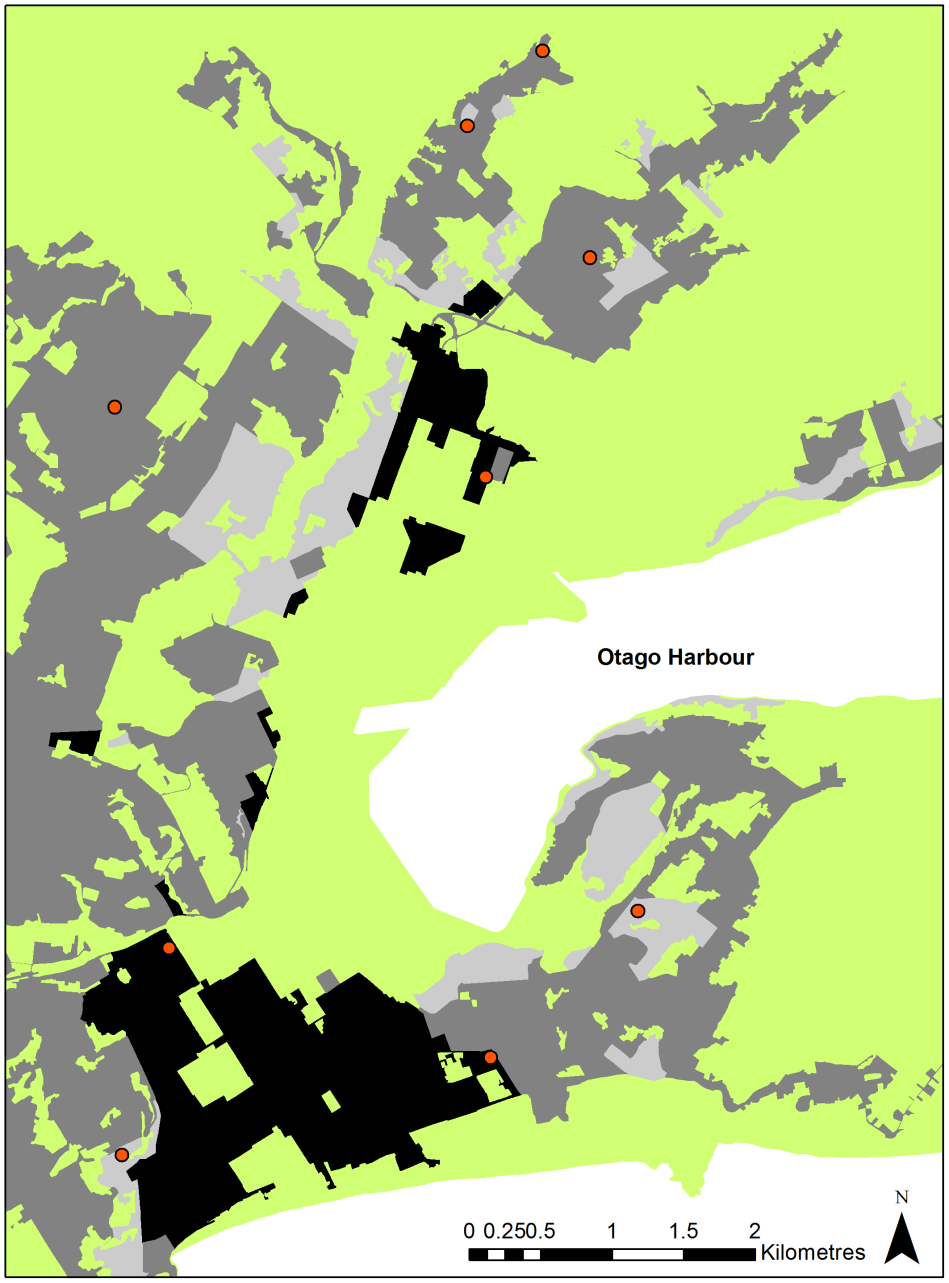
Sampling locations within the suburban environment. Map of the main urban area of Dunedin depicting the sampling locations (orange circles) of stationary GPS collar tests in relation to suburban habitats: Res 1 (light grey); Res 2 (mid-grey); Res 3 (black); and other (light green).

The collars were then evaluated under different environmental conditions within the three suburban habitat types. Three gardens were randomly chosen in each of the three suburban habitats (Figure 1); vegetation in these gardens was evaluated to confirm they fell within the categories defined in Table 1. In each garden, sites representing four categories of sky availability were tested: 0–25%, 26–50%, 51–75%, and 76–100% sky availability (n = 36 sites; 4 sites per garden). Sky availability was assessed using a convex spherical densiometer, which provides relative estimates of percent coverage [37], with coverage including both vegetation and built structures. To determine the impact of buildings on FSR and LE, the distance to the nearest house from the stationary collar was measured at each of the four sites within each garden: distances ranged from 4 m to 34 m and were equally distributed throughout the four sky availability categories. The collars were left at each of the four sites within a garden for 24 hours on a 30 cm high block representing the height of possums when on the ground. For optimal reception of signals from satellites, the GPS collar was placed with the antennae pointing directly upright [5], [12]. The ‘true’ geographic co-ordinates of each site were determined using the average of five locations recorded from a Trimble R7 GNSS using only co-ordinates that were obtained with more than seven satellites present, with precision criteria of <0.015 m horizontally.

### Data Analysis

The FSR for each site was calculated by dividing the number of collected fixes for each site by the maximum number of fixes expected for a 24 hour period (97 fixes). LE was calculated for each collected positional fix by calculating the Euclidean distance between each of the collected positional fixes and the corresponding ‘true’ collar location determined using the Trimble R7 GNSS as follows:
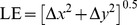
where 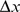 and 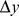 are the differences between the collected and the ‘true’ x- and y co-ordinates respectively [16].

Outlier LE values, which occur in all GPS receivers [8], were identified as fixes falling outside of three times the standard deviation of the mean LE value for each site, and were subsequently removed from modelling. The root mean square (RMS) of the LE (LE_RMS_) was then calculated within each vegetation and sky availability categories, and globally for all sites as a measure of the average location error [38]:
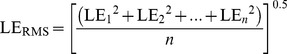



The LE_RMS_ estimate can assist in the selection of buffer sizes around collected positional fixes in resulting spatial analyses for data collected from GPS collars deployed in field trials on animals. Additionally, the arithmetic mean (µLE) and median (µ_1/2_LE) of the LE under each sky availability class were calculated for comparison with previous studies. One-way ANOVAs were performed to determine if the FSR and LE differed between gardens within the three suburban habitats. A two-way ANOVA was performed to determine if LE differed between type of fix (2-D, which is calculated from three satellite signals, or 3-D, which is calculated from four or more signals) due to the possibility that environmental conditions might affect the proportion of 3D fixes, which could have consequences for precision) and sky availability.

A model selection approach was used to identify the predictor variables hypothesised *a priori* that have the greatest impact on FSR and LE separately. For both analyses, data were screened using a Spearman pairwise correlation coefficient (|r| >0.6 cut-off value [39]) to avoid correlated predictor variables within the same model. This test revealed that HDOP and the number of satellites were correlated (Spearman’s pairwise correlation test; r = −0.6, p<0.0001), thus these two variables were not placed together in the same model.

The influence of sky availability, vegetation complexity (as a function of suburban habitat type), and distance to the nearest building on FSR was assessed using fixed effects logistic regression to model the probability of successful locations per site [6]. Eight models representing alternative hypotheses, including the null and global models, were fitted to the standardised data.

LE was modelled using the same standardised environmental predictors as FSR (vegetation complexity, sky availability, and distance to the nearest building), as well as technical predictors including HDOP and the number of satellites present for each positional fix. Linear Mixed Models (LMMs) were used [40] to model the global and null models and all combinations of predictor variables, with models including a random intercept for site to control for the non-independence of the collected positional fixes at each site [41], [42]. Additionally, to evaluate the differences in the generated location errors (which were logarithm transformed to meet the assumption of normality) between 2-D and 3-D positional fixes, linear regression analysis was performed.

For both analyses, model selection was based on the relative difference in Akaike’s second-order corrected Information Criterion (AICc) values, corrected for small sample sizes [43]. The information-theoretic approach involves the development of a set of working hypotheses or models, from which the best model is selected using Akaike’s Information Criterion (AIC), in this case corrected for small samples sizes (AICc). This approach is effective in achieving strong inferences from data analyses [43]. For the LE analysis, we model averaged the coefficients for the fixed effects predictor variables (sky availability, vegetation complexity, HDOP) for the top model set comprising the models with ΔAIC ≤2 [43]. All modelling was performed in the statistical software programme R [44].

## Results

All three collars had a FSR of 1 (100% of the positional fixes were collected) under clear sky at the selected survey mark. There were no significant differences in the accuracy (LE) of the three Sirtrack lightweight GPS collars when simultaneously deployed at the survey mark (F_2,288_ = 1.2, p = 0.27), with the LE ranging between <1.0 m and 106.3 m (median = 16.9 m).

FSRs did not differ between gardens with differing vegetative complexity independent of sky availability (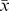 = 90.6%; F_2,33_ = 0.1, p = 0.93). The FSR decreased as the amount of clear sky decreased, ranging from 97% when there was high sky availability, to 81% when there was low sky availability (Table 2). Across all gardens, 64% of the positional fixes obtained were 3-D fixes. The top-ranked model included sky availability only (Table 3); with FSR decreasing with decreased sky availability (increasing canopy cover; coefficient = −0.12, SE = 0.05).

**Table 2:** Fix success rate (FSR ± SD), root mean square of location errors (LE_RMS_), and the mean (µLE ± SD) and median (µ_1/2_LE ± SD) location errors for positional fixes collected from lightweight GPS collars during stationary collar tests under four sky availability classes across three suburban habitat types (n = 36), Dunedin, New Zealand.

					Outliers	
Sky Availability (%)	FSR (%)	LE_RMS_ (m)	µLE (m)	µ_1/2_LE (m)	Mean No. outliers	LE_RMS_ (m)
0–25	81.3±13.6	38.9	29.4±26.6	19.6	5.9±2.8	35.8
26–50	89.6±7.0	30.1	22.6±23.1	16.3	6.4±3.3	29.1
51–75	94.9±1.5	31.8	23.8±19.1	17.8	5.4±3.9	24.7
76–100	96.7±0.6	25.6	17.7±16.2	12.9	7.0±3.0	20.7

**Table 3:** Ranking of models explaining the fix success rate (FSR) obtained by lightweight GPS collars in different suburban habitat types and sky availability classes during stationary collar tests (n = 36), Dunedin, New Zealand.

Model Description	K	AICc	ΔAICc	*w_i_*	Model Likelihood
Sky Availability	3	221.2	0.00	0.50	1.00
Sky Availability+Vegetation complexity	4	223.6	2.35	0.15	0.31
Sky Availability+Distance to buildings	4	223.6	2.36	0.15	0.31
Null model	2	224.7	3.50	0.09	0.17
Sky availability+Vegetation complexity+Distance to buildings	5	226.1	4.86	0.04	0.09
Distance to buildings	3	226.6	5.35	0.03	0.07
Vegetation complexity	3	227.0	5.74	0.03	0.06
Vegetation complexity+Distance to buildings	4	229.0	7.73	0.01	0.02

K = number of parameters; ΔAIC = change in AIC; ***w_i_*** = Akaike weight.

Models were ranked based on the Akaike’s second-order corrected Information Criterion (AICc).

**Figure 2:**
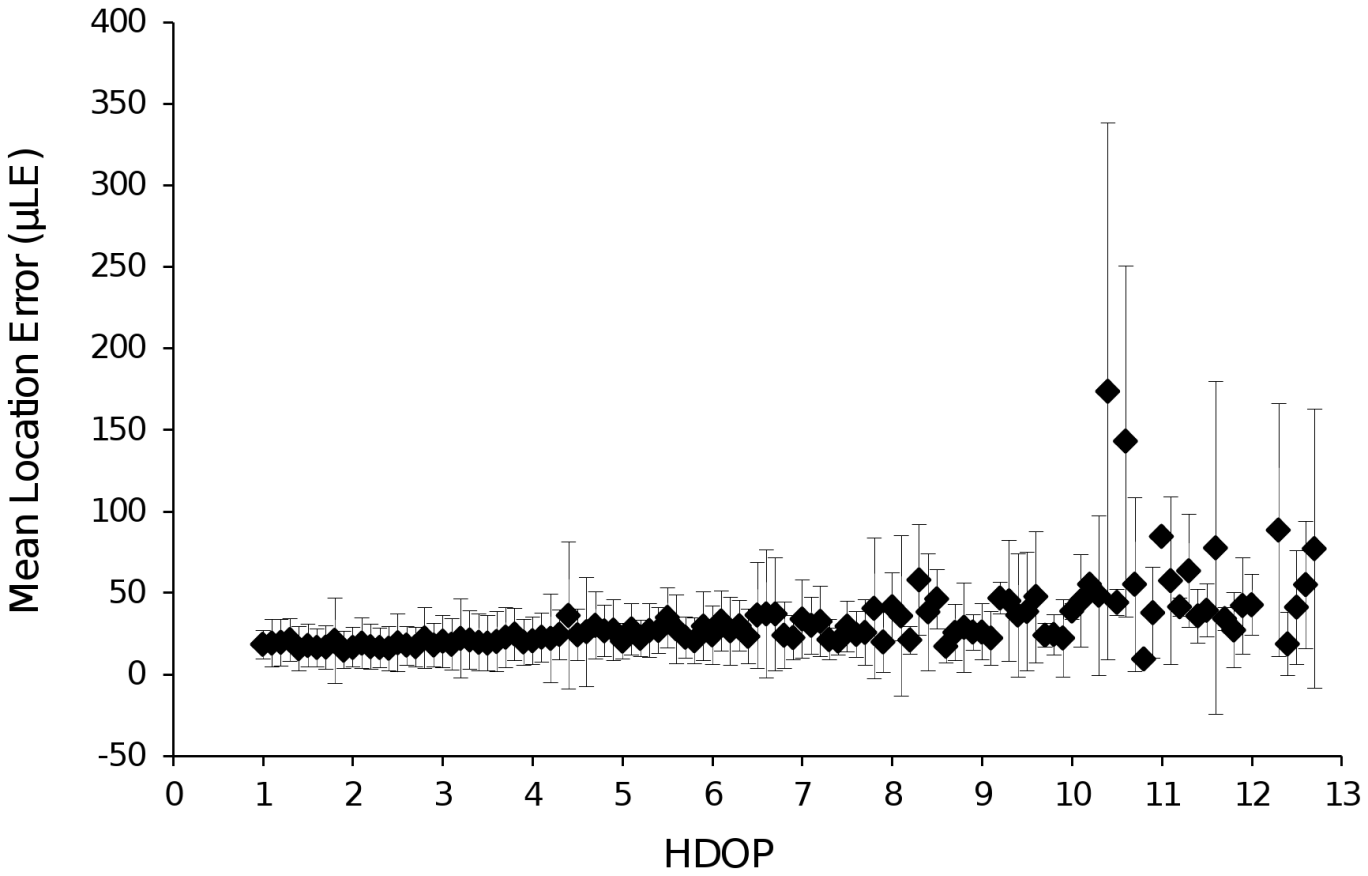
Mean location error (µLE ± SD) for each HDOP value. Mean location error (µLE ± SD) for each HDOP value for lightweight GPS collars across all suburban habitats and sky availability classes, Dunedin.

LE values were significantly different between gardens of differing vegetation complexity (F_2,3243_ = 59.5, p<0.001), with larger LEs obtained in gardens with complex, mature vegetation (Res 1; 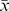 = 33 m) than properties characterised by lawn and flower beds (Res 3; 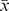 = 28 m). This produced an overall average of 30.1 m for all suburban areas. Additionally, LE tended to decrease with increasing sky availability (Table 2). The calculated µLE also increased with increasing HDOP values, with maximum values being reached for HDOP>10, indicating large, inaccurate values (Figure 2). The large variation in µLE for each HDOP value and associated large standard deviations shows that while LE decreases with increasing HDOP, the ranges also include some positional fixes with similar accuracy to those associated with lower HDOP values (Figure 2).

The magnitude of LE differed depending on whether three satellites (2-D) or four or more satellites (3-D) were available to generate the positional fix, with a significant difference occurring between the type of positional fix obtained (i.e. 2-D or 3-D) and sky availability (F_1,3243_ = 15.0, p<0.001): a higher proportion of 2-D fixes occurred in the 0–25% sky availability class compared to the other three sky availability classes. The LE associated with 3-D positional fixes (LE_RMS = _26.2 m), which accounted for 64% of all positional fixes obtained, were significantly smaller than those associated with 2-D positional fixes (LE_RMS_ = 35.5 m; F_1,3243_ = 39.3, p<0.001). After outlier removal, LE_RMS_ decreased to 21.5 m for 3-D fixes, and 30.7 m for 2-D fixes, indicating that outliers occur in both fix types. A similar number of outliers in relation to LE were obtained within all four sky availability classes (Table 1). Filtering the dataset to remove these outliers improved the LE_RMS_ values by several metres for each sky availability class (Table 1). HDOP values for 2-D fixes ranged from 1.2–12.7 (median = 3.7; 95% of fixes <12.4), while the HDOP of 3-D fixes ranged from 1.0–12.7 (median = 2.3; 95% of fixes <5.0). Additionally, linear regression analysis of the logarithm-transformed LE and associated HDOP values verified that LE values increased with increasing HDOP values (coefficient = 0.05, SE = 0.002, p<2.0^−16^). However, HDOP only explained 14% of the variation within LE (R^2^ = 0.14).

Among the top six models explaining variation in LE, the two that were within Δ AIC≤2 values of each other included the predictors sky availability, vegetation complexity, and HDOP (Table 4). Model averaging of the fixed effects within these top two models showed that HDOP had the strongest effect on LE (coefficient = 0.13, SE = 0.001), followed by sky availability (coefficient = 0.05, SE = 0.03), and vegetation complexity (coefficient = 0.03, SE = 0.03). Therefore, LE increased with increasing HDOP, canopy cover (i.e. reduced sky availability), and vegetation complexity. Distance to buildings, which are characteristic of urban environments, was not present in the top models indicating that this factor was not important in influencing the variation in LE (Table 4).

**Table 4:** Ranking of models explaining the location error (LE) obtained by lightweight GPS collars in different suburban habitat types and sky availability classes during stationary collar tests (n = 36), Dunedin, New Zealand.

Model Description	K	AIC	ΔAIC	*w_i_*	Model Likelihood
Sky availability+HDOP	5	1724.59	0.00	0.61	1.00
Vegetation complexity+HDOP	5	1726.29	1.70	0.26	0.43
Sky availability+Distance to buildings+HDOP	6	1729.40	4.81	0.05	0.09
Vegetation complexity+Distance to buildings+HDOP	6	1729.57	4.98	0.05	0.08
Vegetation complexity+Sky availability+HDOP	6	1730.94	6.35	0.03	0.04
Vegetation complexity+Sky availability+Distance to buildings+HDOP	7	1735.45	10.86	0.003	0.004

K = number of parameters; ΔAIC = change in AIC; ***w_i_*** = Akaike weight.

Models were ranked based on the Akaike Information Criterion (AIC).

## Discussion

This is the first study evaluating the performance and accuracy of lightweight collars in suburban environments. Sky availability had the largest effect on FSR, while LE was affected by sky availability, vegetation complexity within different suburban habitats, and HDOP. Despite the complexity of structures and vegetation within suburban areas, values for FSR and LE were comparable to those obtained in less built-up environments. This produced a mean LE estimate of 30.1 m for suburban habitat types.

Within a suburban environment, FSR increased with increasing sky availability, which is probably due to fewer objects blocking or reflecting satellite signals [5], [45]. For example, high canopy closure can result in a 50% reduction in the FSR [46] due to poor satellite visibility, which also generates a higher proportion of 2-D fixes [10], [31], [47]. We also found evidence that variation in vegetation complexity characteristic of the differing suburban habitat types, and distance to buildings influenced FSR. However, these two habitat variables were relatively less important compared with sky availability. The overall average FSR of 90.6% in this stationary suburban study was slightly inferior to overall average FSRs of stationary studies investigating GPS performance in less built-up habitats with variations in sky availability; e.g. 92.1% in New Zealand farmland habitat [28], 93% and 99% for two brands of collars in forest habitats [11], 92.8% in mountainous habitat [46], and an average FSR of 94.8% from a review of 35 articles [9]. By testing the collars in identical conditions, our study provided evidence that manufacturing differences did not exist between the individual Sirtrack lightweight GPS collars (also see Blackie [27] and Recio et al. [14]).

The accuracy of positional fixes within the suburban environment was dependent on a combination of technical (satellite geometry (HDOP)) and environmental (sky availability and vegetation complexity) variables. Location error increased with decreasing sky availability and increasing HDOP, with LE varying between suburban habitat types of differing vegetation complexity as predicted. However, distance to buildings did not significantly influence LE despite the potential for satellite signals to be reflected off building surfaces.

After filtering outliers, the LE_RMS_ decreased for all sky availability classes, with an overall trend of smaller error values as sky availability increased. It is therefore important to apply a preliminary filter to collected GPS datasets to remove these fixes [8], [14]. The range of average LE values (µLE = 16.0 m to 23.8 m) that were obtained in our study are comparable to those reported in non-urban habitats [9], [10], [11], [14], [31], [46]. This could be a reflection of the suburban habitats containing only low buildings which may not be significantly affecting satellite signals.

LE increased when the number of satellites used to obtain a positional fix decreased. Other studies in a variety of habitats have also documented an increase in error with higher HDOP values (e.g. [7], [15]); higher HDOP values are usually associated with poor satellite configurations, such as when only three satellites are available or when satellites are clustered [46]. Additionally, 2-D fixes, which made up 36% of all fixes in our study, were less accurate than 3-D fixes, and were largely associated with decreased sky availability. This result is slightly higher than proportions obtained in other habitats (30.2% in farmland [28]; 31% in boreal forests [12], and 31.4% in mountainous habitat [46]), and is a reflection of satellite geometry and number of satellites available (e.g. [16], [31], [48]). In less built-up habitats, poor satellite configurations are associated with reduced sky availability caused by dense canopy cover, high terrain, or physical objects masking the sky [13], [15], [18], [19], [20]. Differences in these environmental conditions, which influence the number of satellites available to the GPS device, will also result in differences in the precision of acquired 3-D fixes [14], [20]. In the residential environment, poor satellite configurations due to reduced sky availability are likely to be associated with vegetation cover within individual gardens, particularly in areas containing large proportions of complex, mature vegetation, especially tall trees (i.e. Res 1). However, the results also indicate that accurate positional fixes can be obtained for large HDOP values and for 2-D fixes. Therefore, by using traditional filtering methods, such as discarding 2-D fixes [10] or fixes with an associated HDOP≥9 (e.g. [31]), accurate locations will also be discarded. Additionally, the removal of all 2-D fixes can reduce the dataset significantly. From these results, it is recommended to utilise more recent filtering approaches, which incorporate species-specific assumptions regarding unrealistic speeds and distances travelled between consecutive locations [49] to filter collected datasets from suburban environments.

Distance to buildings was not an important predictor variable affecting the FSR or LE in this study. Our finding is similar to results from Rose et al. [29] who documented that LE decreased significantly in an area with high storage buildings, but was similar in all other urban locations tested. They also found that FSR decreased as the amount of open sky decreased [29], although there was no mention of whether sky obstruction was due to building presence or vegetation. Therefore, the non-significant result reported in this study may be a reflection of the density and height of the surrounding buildings. Our research was performed in private gardens of low-rise suburban areas of the city (i.e. one to two storey buildings) as these areas typically make up a large proportion of urban landscapes: 36% in Dunedin [33], and can support significant populations of wildlife [34], [36], [50], [51]. The low heights of buildings within these suburbs may not be high enough to reflect or block satellite signals. The opposite may be true for high-rise areas of the city, for example the Central Business District (CBD) and/or industrial sectors, which contain a greater proportion of tall buildings.

Our study only evaluated stationary lightweight GPS collars and did not incorporate the impact that animal behaviour and movement might have on FSR and LE values. Signal acquisition of collars has been shown to be affected by animal behaviour and body position in that different activities, particularly denning and foraging, can result in the orientation of the antennae being horizontal relative to the sky, leading to lower FSRs and number of 3-D fixes [5], [52]. For example, D’Eon [30] reported that the main source of lost data (i.e. a low FSR) is associated with animal activity, while Lewis et al. [31] reported a reduction of 11% in the FSR when collars were deployed on live animals. However, in stationary tests the antenna is always directly vertical to the sky, maximising signal reception, as antennae on collars are designed in an upright position to optimise signal acquisition [5], [7], [12], [20], [23], [53]. Researchers should therefore consider the behaviour of their study species as well as environmental and technical factors when evaluating the performance of their collars in the field. Possums forage both vertically and horizontally, which may affect the FSR and LEs which is speculated to improve with increased positional height in trees due to less blockages interfering with the satellite signals. By evaluating collar performance close to the ground, we took a conservative approach in calculating collar error, as error is expected to be greater at ground-level due to canopy cover. Additionally, by developing an error estimate independent of animal behaviour for suburban environments, our results have a general applicability for use in other studies conducted in similar environments.

### Management Implications

Determining the performance and accuracy of GPS collars in a study location is important for making accurate conclusions from the collected spatial data and appropriate decisions regarding species and habitat management. We found a relatively large LE (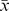 = 30.1 m) was associated with lightweight GPS collars within suburban habitats. Location error of this size is less important for determining home range sizes, but is likely to have significant impacts on habitat and resource selection analyses, and should be accommodated through the use of buffers reflecting habitat-specific LEs around each positional fix. However, because urban environments are highly heterogeneous at a fine scale, large buffers based on LEs can include multiple habitats or resources, and it can be difficult to accurately identify which habitats and/or resources animals are using. Additionally, overlapping buffers, due to their large size, can be problematic when trying to differentiate the predictive landscape features of available and used areas [54]. Unless the error of GPS devices can be reduced through better technology, our results suggest that conclusions about resource and habitat selection in heterogeneous suburban environments should be made with caution, and other techniques that are not reliant on buffer sizes, such as Brownian bridges [51], [52] that incorporate location error directly into the analysis, should be considered.
